# Can you See What We See? African American Parents’ Views of the Strengths and Challenges of Children and Youth Living with Adversity

**DOI:** 10.1007/s11121-022-01469-y

**Published:** 2022-11-28

**Authors:** Oscar A. Barbarin, Nikeea Copeland-Linder, Michael Wagner

**Affiliations:** 1 African-American Studies, University of Maryland, College Park, MD USA; 2 Kennedy Krieger Institute, Baltimore, MD USA; 3 Johns Hopkins University, Baltimore, MD USA

**Keywords:** Adversity, Family stress, Behavior problems, Positive youth development, Resilience

## Abstract

A premise of positive youth development is that social competencies can develop in adversity and co-exist with problem behaviors. This research tested whether African American youth ages 9–17 who had experienced significant family stressors would form groups that displayed combinations of adversity, problem behavior, and strengths. Parents of a nationally representative sample of African Americans children were interviewed on child difficulties and strengths as part of the CDC’s 2019 National Health Interview Survey. About 15% of the national sample of African American youth had experienced violence or parental incarceration, depression, or drug abuse. Latent class analysis (LCA) using indicators of adversity and strengths identified four distinct classes. Class 1 included youth who experienced multiple adversities, exhibited few strengths, and were high in behavior problems. Members of both classes 2 and 3 were more likely to experience parental incarceration but exhibited altruism. Class 3 also experienced parental mental health problems. Members of class 4 had the highest exposure to violence but were comparatively high in altruism and affability. Regression analysis revealed that the groups differed from one another on emotional health but not on physical health controlling for age and gender. These findings support a focus by mental health prevention programs on building on the strengths of children growing up in adversity.

African American children disproportionately grow up in chronic poverty and face multiple forms of adversity over the course of their development. They are negatively affected not only by the adverse events they experience directly but also indirectly by the toxic stressors experienced by their parents. The increased stress experienced by parents can negatively affect the mental health of their children by compromising the parent’s ability to care for and support their children. Thus, adversity in its many forms can imperil the well-being of children and youth by placing them at increased risk of mental and physical health problems (Shonkoff et al., 2012).

## The Context of Adversity Among African American Youth

Economic inequality among African American families is a legacy of racial discriminatory practices and policies in the USA (Bailey et al., 2017). The family stress model has been used to explain how economic hardship impacts children indirectly by increasing the strain experienced by their caregivers (e.g., Gard et al., 2020). Economic hardship increases parents’ psychological stress which may strain the parent–child relationship and negatively impact parenting practices, and this may ultimately lead to maladaptive youth outcomes. Other studies suggest that poverty gets “under the skin” by exposing youth to an increase in stress and trauma that causes dysregulation of the stress response system and affects the development of brain structures involved in regulating emotions, impulse control, and executive functions (Blair & Raver, 2012).

### Exposure to Violence and Youth Outcomes

African American youth are disproportionately affected by violence as victims and witnesses (Sheats et al., 2018). Killings of unarmed African American youth have drawn media attention to disparities in police brutality experienced by African Americans. In addition, African American youth often witness and experience violence due to their disproportionate representation in neighborhoods characterized by disadvantage that may expose them to violence in multiple contexts including family, community, and school (Mrug et al., 2008). Between 50 and 96% of urban youth have witnessed some form of community violence (cited in Gorman-Smith et al., 2004).

Exposure to violence has been linked with several mental health problems, including depression, anxiety, and posttraumatic stress symptoms and disorder, and suicidal ideation and attempt among youth (e.g., Fowler et al., 2009). Research on witnessing violence indicated that having a closer relationship to the victim of violence is associated with more mental health symptoms (Kennedy & Ceballo, 2014). In addition, several studies have linked exposure to violence with increased health risk behaviors, including aggressive and violent behavior, substance use, and sexual risk taking (e.g., Voisin & Takahashi, 2021).

### Parental Mental Illness and Substance Abuse

Parental mental illness and substance abuse are major public health issues affecting the lives of children. Approximately 18% of US parents suffer from a mental illness, and 3.8% suffer from a serious mental illness (Stambaugh et al., 2017). In addition, 8.4% have a substance use disorder (Lipari & Van Horn, 2017). Approximately 12.3% of children aged 17 or younger reside with at least one parent with a substance use disorder (Lipari & Van Horn, 2017).

African Americans have lower rates of lifetime and past year psychiatric disorders than whites (Miranda et al., 2008). However, when they do experience mental illness, they tend to have a more severe and persistent course of the disorder (Breslau et al., 2005). Longitudinal studies provide evidence for associations between maternal depression and emotional outcomes across domains and age ranges for youth, including internalizing disorders, poor social competence in school years, and increased risk of depression during adolescence. In a systematic review, paternal depression was associated with children’s behavioral and developmental problems, poor school performance, and risk of developing psychiatric disorders (Gentile & Fusco, 2017). In addition, a recent meta-analysis conducted by Pierce et al. (2020) revealed significantly higher rates of asthma and injuries among youth of parents with a mental illness.

### Parental Incarceration

There are approximately 2 million people in the USA in prisons or jails (National Research Council, 2014). Estimates indicate that 2.7 million children experience parental incarceration on any given day and over 5 million children (7% of US children) have experienced parental incarceration at some point in their lives (Murphey & Cooper, 2015).

The increase in mass incarceration has disproportionately affected African American families due to the “war on drugs” and strict sentencing over minor crimes that resulted in the targeting of and stereotyping African Americans as drug addicts ( Bailey et al., 2017). In addition, African American men are more likely to be sentenced, and they serve longer sentences than their white counterparts for the same offense (Morsy & Rothstein, 2016). The discriminatory arrests and sentencing of African American males have resulted in a disproportionate number of African American youth suffering from parental incarceration. An African American child is six times as likely as a white child to have an incarcerated parent (Morsy & Rothstein, 2016).

Parental incarceration is a traumatic stressor with lasting consequences (Foster & Hagan, 2013). Studies show that between 22 and 41% of children with incarcerated parents have witnessed their parent’s arrest (Phillips & Zhao, 2010). Moreover, chronic stressors associated with parental incarceration include prolonged separation from a parent, strained family relationships, economic instability, and stigma. Parental incarceration can have detrimental effects on child well-being in various domains including emotional, social, and health across the life course (Foster & Hagan, 2013). It has been associated with internalizing and externalizing symptoms for youth. In a sample of 11 to 17 year olds, youth who were ever exposed to parental incarceration were more likely to have their caregiver report attention, internalizing, externalizing, and total problem behaviors, controlling for demographic characteristics of the youth. These youth were also more likely to have poor attention and externalizing problems, above and beyond socioeconomic characteristics (Boch et al., 2019).

### Prosocial Competencies in the Context of Adversity

Past research on African American children has revealed important insights about the risks of impairment associated with family adversity. However, much less is known about the extent to which social competencies arise under adverse conditions. Investigations of the cultural and social assets available to African American youth provide a basis for optimism about the possibility of positive developmental outcomes in spite of adversity (Barbarin et al., 2020). In the context of adversity, youth may draw upon family, community, and cultural resources that enable them to develop heightened cognitive, affective, and social competencies that are specifically useful and adaptive in difficult environments (Frankenhuis & de Weerth, 2013).

Positive youth development (PYD) provides a framework for understanding how these psychosocial strengths can develop under conditions of adversity and can co-occur with problem behavior. According to the PYD framework, youth can develop social competencies with the help of family and community no matter what adversities they experience. In the case of African Americans, it also includes relying on traditional cultural resources such as religious beliefs and support from extended family networks (Larson, 2000; Lerner et al., 2003). Within the PYD framework, caring, connection, confidence, competence, and character are especially important social competencies. (Lerner et al., 2003; Thulin et al., 2022). They have been associated with the prevention of adolescent pregnancy, drug abuse, and juvenile offending (Benson et al., 2005; Lerner et al., 2009). Moreover, the presence of conduct problems does not rule out the possibility that these prosocial competencies will develop (Tolan et al., 2003).

This paper examines whether two specific dimensions identified by PYD (viz., connection and caring) co-occur with adversity and problem behavior. Terms such as affability, agreeableness, sociability, and civility have been used in research on the concept of connection (Crowe et al., 2018). Affability refers to the concept of connection, and altruism is used to denote caring. Affability is a proclivity toward friendliness that facilitates getting along with others. It includes children’s willingness to cooperate and inhibit the behaviors such as fighting and arguing that damage sociability. Affable children are more likely to attract and engage others who might offer assistance needed to cope with and overcome distress related to adversity (Busby et al., 2009). It has been associated with positive peer relations (Jensen-Campbell & Graziano, 2001), reductions of interpersonal conflict and prejudice (Sibley & Duckitt, 2008), and high quality of intimate dyadic relationships (Busby et al., 2009). Children suffering the most extreme conditions of poverty and violence display levels of affability similar to levels observed among children living in more propitious environments (an der Merwe & Dawes, 2000). Altruism is a related social competence. It refers to caring about, displaying generosity, and helping others. It is associated with better emotional functioning, ability to cope with stress, and reduced aggression (Thulin et al., 2022). Tashjian et al. (2021) demonstrated that altruism moderated the positive effects of acts of kindness on increasing positive affect, decreasing negative affect, and reducing stress. Altruism was associated with lower levels of violence and substance abuse among children who grew up in adversity (Thulin et al., 2022).

### The Present Study

The primary aim of the present study was (a) to identify distinct profiles of children and youth based on a combination of adversity (e.g., parental incarceration, violence exposure, and parental mental illness/substance use), social competencies, and conduct problems. In addition, it examined the psychological and physical health status of these groups of children and adolescents. Previous research has demonstrated how that family stressors are linked to problem outcomes but not how the two combine with social competence. For example, parental incarceration and experience of violence have been linked to conduct problems; parental mental illness and substance abuse to poor social skills and health; and all three to emotional problems. Although we expected that our findings on the relations between stress and problems would be consistent with prior research, we had no basis for predicting the profiles the would combine all three.

## Method

Data for this study were from the National Health Interview Survey (NHIS) collected in 2019 by the US Census Bureau for the Center for Disease and Control.

### NHIS Survey Design and Procedures

The National Health Interview Survey (NHIS) is a cross-sectional household interview survey of the noninstitutionalized US population conducted each year. The NHIS sampling plan uses a multi-stage area probability design that began with 428 primary sampling units (PSUs) drawn from approximately 1900 geographically defined PSUs that covered the 50 states and the District of Columbia. African American, Asian, and Latinx households were oversampled. Interviewers employed and trained by the US Census Bureau administered the NHIS computerized questionnaire and entered responses directly during the interview. One household respondent supplied basic demographic and relationship information about all household members. One child in each household was randomly selected for the study. Information was provided by an adult knowledgeable about the child’s health and functioning, most often a parent or guardian.

### Participants

The data from a subset of African American children and adolescents ages 9 to 17 collected in 2019 (weighted *N* = 5,930,560) was used in the present analysis. Children younger than 9 were excluded. Females made up 51% of the sample and adolescents 13–17 constituted 46% of the sample.

### Measures

The child’s parent or primary caregiver completed all measures.

### Family Stressors

Respondents indicated whether the target child experienced parental incarceration, drug use, mental illness, and victimization or witnessing violence. The response format was 1 = yes or 0 = no.

### Material Deprivation

An index was formed by summing the dichotomized indicator of income poverty, i.e., household income below 150% of Federal poverty guideline (0 = not poor, 1 = poor); food insecurity, i.e., inadequate food support, skipping or cutting meals altogether (0 = no 1 = yes), and financial worry; and i.e., worries about and difficulty paying bills and (0 = none 1 = any) yielding scores ranging from 0 to 3. A dichotomized version was created in which a household was categorized as materially deprived if they were positive on at least one of the 3 material deprivation indicators.

### Strengths and Problems

Information on strengths and conduct problems was collected using the Strengths and Difficulties Questionnaire, ([SDQ], Goodman, 1997). Items from the SDQ used a 3-point rating scale (0, 1, 2) in which parents indicated the extent to which a statement was true of the target child (0 = not true, 1 = somewhat true, 2 = definitely true). Each factor-analytically derived scale consisted of five items. Items were coded so that high scores reflected competence and strengths. Affability was operationalized using the peer problems scored in the direction of friendliness and likeability. Sample items include “liked by other children” and “solitary, prefers to be alone” (reverse coded). Altruism was measured using items from the prosocial subscale of the SDQ that referred to empathy, sharing, and caring for others. Sample items included “is kind and considerate” and “offers to help others”. Conduct problems were measured using items from the SDQ conduct scale, and it assessed problem behaviors such as losing temper, fighting, cheating, and lying. Sample items include “steals from home, school or elsewhere” and “is well-behaved” (reverse coded).

### Health

These items described the functional status of children and youth using the World Health Organization’s International Classification of Functioning, Disability, and Health (ICF) as a conceptual framework. The questions identified functional limitation of children.

#### Emotional Health

Two emotional health items were based on the World Health Organization assessment of the functional status of children and youth using the International Classification of Functioning, Disability, and Health (ICF) as its conceptual foundation. Anxiety was a single item that asked how often the child seems very anxious, nervous, or worried. The 1–5 scale is as follows: 1 = daily, 2 = weekly, 3 = monthly, 4 = a few times a year, or 5 = never. Sadness was a single item that asked how often the child seems very sad or depressed. It used a similar 5-point scale.

#### Physical Health

The health status of sample children was assessed using a 5-point scale developed and used by the WHO. Parents reported whether the child’s health is excellent, very good, good, fair, or poor. This item was scored so that lower scores indicated better health.

### Analysis Plan

To explore the relationship among family stressors, psychosocial competence, behavior, and health, we selected and analyzed data from children ages 9–17 who experienced at least one of the four family stressors assessed in the NHIS study: viz., parental mental health problems, incarceration, or substance abuse or witnessing or experiencing violence. We excluded children under nine from the analyses because of differences in cognitive competences relevant to our measures. The LCA included pre-teens and teens because by age nine children have the capacity for executive function, social awareness, moral reasoning, self-regulation of emotion and behavior, prosocial behavior, empathy, positive peer relations, and altruism. Moreover, longitudinal studies of behavior of African American reveal that inflection points in the developmental trajectories of behavior problems occur over the transition from late primary to early middle school (Barbarin et al., 2019). In addition, by age nine, children have the capacity for social skills and empathy that undergird the psychosocial strengths measured in this study. However, because age and gender are related to depression and anxiety, we controlled for the effect of gender, age, and its interaction with the classes in the regression analyses.

We used latent class Analyses to examine whether African American youth form distinct groups on the basis of a combination of adversity, problems, and strengths. The adversity indicators used in the analyses were whether youth experienced violence or had parents affected by incarceration, drug abuse, and mental health problems. Three other indicators of child functioning were included: altruism, affability, and conduct problems. Because the measures of adversity were dichotomous, the LCA utilized dichotomous versions of the strengths and problems for consistency and because of the bi-modal distribution of the indicators. The four adversity items were dichotomous, and the strengths and problems were effectively dichotomous since over 50% of responses were concentrated in one response category.

Latent class analyses were conducted utilizing dichotomized (yes/no) versions of each of the sources of adversity, child social competencies, and conduct problems. The latent class model examined the relationships between these eight observed variables by assuming that these relationships are explained by an unobserved latent categorical variable. We also assumed conditional independence (Muthén, 2001). This procedure produced measurement and structural parameters. These were used to estimate the likely class membership. Given a set of **r** dichotomous items **u** that result in **K** classes, the marginal probability for item **j** being equal to 1 is given by the formula:

$$\mathrm{P}({u}_{j}=1) = \sum\limits_{k=1}^{K}P(\mathrm{c}=\mathrm{k})\mathrm{P}({u}_{j}=1|\mathrm{c}=\mathrm{k})$$

where **c** is the latent class variable and **k** is a class within c. The structural part of this expression, *P*(*c* = *k*) describes the observations in terms of the probability of class membership. $P({u}_{j}=1|c=k)$ describes the measurement part of the model or the probability of endorsing the item conditional on the class or the conditional item probability. The joint probability of all items assuming conditional independence is:

$$\begin{aligned}P({u}_{1},{u}_{2},...,{u}_{r})&= {\sum\nolimits_{k=1}^{K}}P(\mathrm{c}=\mathrm{k})\mathrm{ P}( {u}_{1}|\mathrm{ c}=\mathrm{k})\\&\mathrm{ P}({u}_{2}|\mathrm{ c}=\mathrm{k}) \dots .\mathrm{ P}({u}_{r}|\mathrm{ c}=\mathrm{k})\end{aligned}$$

These probabilities are then used to compute the posterior probabilities used in estimating most likely class membership:

$$P(c = k|{u}_{1},{u}_{2}, ... , {u}_{r})=\frac{p\left(c=k\right)P({u}_{1}|c=k)P({u}_{2}|c=k)..P({u}_{r}|c=k)}{P({u}_{1},{u}_{2},...,{u}_{r})}$$

To identify the sample’s profiles that integrated strengths and stressors, we estimated a series of models beginning with one class and continued until the model with the best fit was identified. We selected a model based on several criteria: a)interpretability within the stress and strength framework, and b) statistical criteria that included smaller AIC and BIC, and entropy and the lowest average latent class probability greater than .80. To test the model validity, we conducted regression analyses that examined the relationship between the classes and depression, anxiety, and physical health ratings. Measures of emotional and physical health were dependent variables in the regression, and dummy variables created for the classes using class 1 as the index were the independent variables. Child age, sex, their interaction, and the interaction of adversity indicators with age were included as control variables in the regression analyses.

## Results

Slightly over 15% of the total African American children in the NHIS sample (*N* = 897,290) had at least one family stressor, and many had more than one. Almost half (48%) of the group reported some form of material deprivation, and about one-third lived in households that reported financial worries and living on incomes less than 150% of the federal poverty guideline and 41% reported food insecurity. A majority resided in the south (60%) and about 16% each resided in the northeast and the Midwest. About two-thirds of the sample lived in large central metropolitan areas, and most rented their homes. The sample used in the analyses consisted of children and youth ages 9 to 17 and children who experienced at least one of the four household stressors: 28% experienced parental substance abuse; 35%, mental health problems; 41%, parental incarceration; and 50% experienced violence. In addition, 27% were rated as affable, 55% as altruistic, and 56% had conduct problems.

**Fig. 1:**
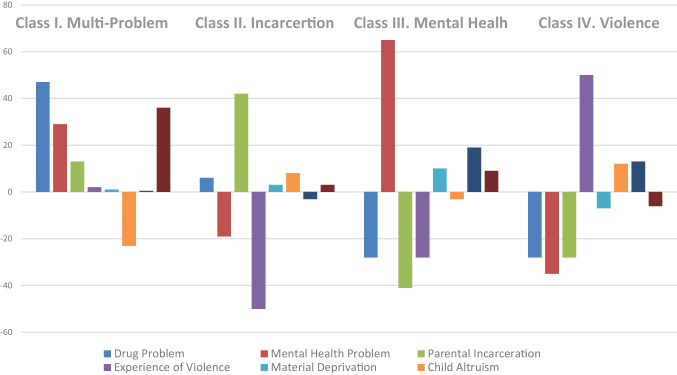
Family problems, child strengths, and problem indicators by class

Solutions were generated for up to five classes. Table 1 presents the model fit statistics. This was the preferred model even though the BIC statistic had started increasing in the 3-class solution. The Pearson Chi-square test of model fit for the binary and ordered categorical indices suggest that the resulting four-class solution was a good fit for the data (*χ*^2^ (220) = 229.96, *p* = .309). The likelihood ratio Chi-square also suggests that the model fits the data well (*χ*^2^ (220) = 174.29. *p* = .990). The indicator of entropy was .87. In addition, the classification probabilities for the most likely latent class membership supports the accuracy of case assignment to categories for each of the 4 latent categories: .80 for class 1, .93 for class 2, .98 for class 3, and .99 for class 4. For the entire sample experiencing at least one family stressor, the mean probability for drug use was 28%; for depression, 35%; for incarceration, 41%; for violence, 50%; for material deprivation, 42%; for altruism, 55%; for affability, 27%; and for conduct problems, 76%. This class solution involved distinct combinations of adversity, strengths, and problems as expected. We labeled each class using the dominant form of adversity and the strengths. Figure 1 presents the relative proportion of cases in each class that were a yes on the specific indicator. Data for the relative proportion was zero-centered and represented each class in term of its difference from the mean (see Fig. 1).

**Table 1:** Model fit statistics for one- to five-class specification of latent class analysis model

Number of classes	LLH	Change in BIC	Pearson χ^2^, *p* value	Likelihood ratio χ^2^, *p* value	Entropy
1	− 708.0	–	.0000	.0122	–
2	− 678.7	14.3	.0041	.4287	.808
3	− 659.9	− 6.3	.0292	.8866	.819
4	− 645.3	− 15.0	.3088	.9898	.865
5	− 634.4	− 22.4	.7297	.9991	.875

### Description of Latent Classes

Class 1 multi-problem/conduct disordered/low strengths (Weighted *N* = 208,598 or 25%). Relative to children in the other classes, children in this class had the highest probability of experiencing parental drug use and the second highest probability of experiencing violence and parental mental illness and incarceration. They were comparatively low on ratings of psychosocial competence. There were equal numbers of males and females, but 68% are pre-teens (ages 9–13) and were living in poor households (< 150% of poverty line).

Class 2 incarcerated parent/altruistic (weighted *N* = 247,615 or 27%). Children in this class had the highest probability of experiencing parental incarceration. Compared to the other classes, they were high on altruism (48%) but were also high with respect to conduct problems. This class was made up of equal numbers of males and females and equal numbers of pre-teens and teens.

Class 3 depressed parent/altruistic (weighted *N* = 148,584 or 14%). Class 3 had the fewest children. Children in this class had a 100% probability of experiencing parental depression or some other mental health problems. Children of parents with mental illness are more likely than other groups to experience poverty. They were less likely to have conduct problems (38%), were more likely to display affability (47%), and were moderately high on altruism. In addition, they were more often males (61%), pre-adolescent (68%), and living in poverty.

Class 4 violence exposed/altruistic/affable (weighted *N* = 292,493 or 33%). Class 4 was the largest class. Children in this class have a 100% probability of experiencing or witnessing violence. They were more likely than other groups to display both altruism (51%) and affability (41%) and at the same time rated as having conduct problems (58%). There were more females (59%) than males and more likely to be teens (60%).

### Class Differences in Emotional and Physical Health

None of the models testing the effects of interactions among child gender, age, and adversity (parental incarceration, parental mental health problems, or child experience of violence) were significant. In hierarchical regression model, step 1 for depression was significant, *F*(3130) = 4.576, *p* < .01. In regression model, step 2 for depression was also significant, *F*(5128) = 2.796, *p* < .05, but the *r*-square change was not significant *F*(2128) = .210, *p* = n.s. The standardized beta coefficients for each of the classes were significant with and without controls for age and sex. These results revealed that classes 2, 3, and 4 were significantly less depressed than class 1, the multi-problem group (see Table 2).

**Table 2:** Youth depression: hierarchical multiple regression predicting depression scores by class, age, and sex of youth

Variable	*B*	95% confidence interval		SE B	*β*	Δ *R*^2^
		Lower bound	Upper bound			
Step 1						.096^b^
Constant	1.300	.930	1.670	.187		
Class 2	− .895	− 1.392	− .397	.252	− .377^c^	
Class 3	− .573	− 1.142	− .004	.287	− .200^a^	
Class 4	− .700	− 1.178	− .222	.241	− .312^b^	
Step 2						.003
Constant	1.376	.913	1.840	.234		
Class 2	− .893	− 1.395	− .391	.254	− .376^c^	
Class 3	− .577	− 1.150	− .004	.289	− .201^a^	
Class 4	− .685	− 1.171	− .200	.245	− .305^b^	
Youth sex	− .111	− .465	.243	.179	− .052	
Youth age	− .037	− .410	.336	.188	− .017	

*Class 2*, incarcerated/altruistic compared to multi-problem /conduct problem. *Class 3*, mental health/altruistic compared to multi-problem/conduct problem. *Class 4*, violence experience/altruistic/affable compared to multi-problem/conduct problem

^a^*p* < .05

^b^*p* < .01

^c^*p* < .001

In hierarchical regression model, step 1 for anxiety was significant, *F*(3129) = 2.806, *p* < .05. In regression model, step 2 for anxiety was also significant, *F*(5127) = 2.399, *p* < .05, but the *r*-square change was not significant *F*(2127) = 1.739, *p* = n.s. Class 2 was significantly lower on anxiety than the multi-Problem group, but classes 3 and 4 were not (see Table 3). None of the hierarchical regressions models for physical health was significant.

**Table 3:** Youth anxiety: hierarchical multiple regression predicting depression scores by class, age, and sex of youth

Variable	*B*	95% confidence interval		SE B	*β*	Δ *R*^2^
		Lower bound	Upper bound			
Step 1						
Constant	1.448	.987	1.910	.233		.061^a^
Class 2	− .881	− 1.497	− .264	.312	− .309^b^	
Class 3	− .585	− 1.287	.118	.355	− .170	
Class 4	− .382	− .974	.210	.299	− .141	
Step 2						.025
Constant	1.680	1.111	2.248	.287		
Class 2	− .889	− 1.504	− .274	.311	− .312^b^	
Class 3	− .608	− 1.308	.091	.354	− .177	
Class 4	− .350	− .945	.245	.301	− .130	
Youth sex	− .404	− .835	.027	.218	− .158	
Youth age	− .040	− .494	.413	.229	− .015	

*Class 2*, incarcerated/altruistic compared to multi-problem /conduct problem. *Class 3*, mental health/altruistic compared to multi-problem/conduct problem. *Class 4*, violence experience/altruistic/affable compared to multi-problem/conduct problem

^a^*p* < .05

, ^b^*p* < .01

^c^*p* < .001

## Discussion

The LCA and regression analyses used here examined relationships among familial stressors, psychosocial strengths, conduct problems, and health outcomes of African American youth exposed to adversity. We found that the latent class analyses provided conceptually coherent profiles organized around one or more of the household stressors and child strengths. We expected that for African American children and youth psychosocial sequelae of adversity in the form of problem behavior would co-exist and co-develop along with psychosocial competencies such as altruism and affability. The results support the claim that prosocial competencies often co-occur with conduct problems under conditions of adversity as suggested by Tolan et al. (2003) with one major exception. Under conditions of multiple family stressors, social competencies were much less likely. This is an important exception that presents a difficult challenge for programs working on behalf of children whose families are beset by multiple problems. The multi-problem group faced many different sources of adversity. Their profile was consistent with research on effects of cumulative stress in that they had the poorest outcomes (Sameroff & Seifer, 1983).

For the other groups, the results are consistent with the findings of an der Merwe and Dawes (2000), and they support PYD positive youth development principle of co-existence of psychosocial competencies and problems. In three out of the four classes, psychosocial competencies were found at the same time that difficulties were reported.

Consistency of our findings with prior research on the sequelae of family stress and problems varied by family stressor. For incarceration and violence, our results confirm previously reported relations with conduct and emotional difficulties (Boch et al., 2019; Foster & Hagan, 2013; Lambert et al., 2008; Voisin & Takahashi, 2021). However, children whose families experience parental mental health or substance abuse relative other groups were not more likely to manifest poor social skills or difficulties with emotional or physical health. This does not suggest that these difficulties were absent in the lives of these children but that they were better off on these issues than the other groups. Children experiencing parental mental health problems or violence had profiles that were most consistent with the PYD premise of co-existing problems and strengths. Adopting a strength perspective such as PYD to build strengths could be very effective for these groups.

The design of mental and behavioral health prevention effort should consider how to build on the strengths of children and youth deemed to be at risk as a consequence of adversity. Children with high levels of conduct problems related to family stressors might respond better to strategies that draw on their ability to get along with others or by appealing to their empathy and willingness to help others. Prevention programs might be more successful if their efforts increase strength as much as reducing problem behavior. Similarly, the criteria used to evaluate prevention programs should extend beyond reduction of problems to building social competencies. Prevention programming based on PYD offers such an approach. It is a multi-component, prosocial approach that engages youth within their communities, schools, organizations, peer groups, and families; encourages parental monitoring and support; involves youth in serving others; and builds on youth leadership skills (Benson et al., 2005). Prosocial strengths can be assets that help programs neutralize corrosive effect of adversity (Lerner et al., 2003). Similarly, interventions in educational systems based on restorative justice principles offer an excellent example for programs that embrace a more positive, optimistic, and humane view of children who may be struggling particularly those whose difficulties can be linked to economic and social disadvantage arising from systemic racism and structural inequalities (Gregory et al., 2016).

### Limitations

The results of this study should be viewed as exploratory and suggestive due to several limitations. Interpretability of the LCA was a primary consideration in our choice of models, but a strong case could be made for an alternative model solution on the basis of the small differences among classes 2, 3, and 4. A more parsimonious 2 class model could have been fitted to the data though it would have diminished our understanding of subtle differences among groups of children based on the dominant family stressor. The cross-sectional nature of the data means that it is not possible to draw causal inferences regarding the relationships between classes and health outcomes. Longitudinal studies could address this concern by examining how the combinations of adversity, strengths, and problem behavior at one point predict future health outcomes. The reliance on parent report is another limitation. Parent’s reports of youth strengths and difficulties have well-documented biases. Parents’ reports differ from youths’ report in systematic ways with youth under-reporting conduct problems and parents’ under-reporting internalizing problems (De Los Reyes et al., 2015). More robust designs should use with multi-informant reports of adversity, strengths, problem behaviors, and health outcomes. A final limitation is the use of a classify-analyze approach. This approach is vulnerable to attenuation of estimated coefficients (Bray et al., 2015). This research is just a starting point for understanding how adversity and strengths cohere within African American youth who have experienced significant family stressors. Classes are suggestive and need to be replicated using other data sources. In addition, the relation of classes to health outcomes should be tested using more robust measures of physical and emotional health.

## Data Availability

The data used in this report were taken from a publicly available files for the National Health Interview Survey at the CDC. They can be retrieved from the CDC website: https://www.cdc.gov/nchs/nhis/index.htm.
